# Determination of carcinogenic herbicides in milk samples using green non-ionic silicone surfactant of cloud point extraction and spectrophotometry

**DOI:** 10.1098/rsos.171500

**Published:** 2018-04-11

**Authors:** N. I. Mohd, N. N. M. Zain, M. Raoov, S. Mohamad

**Affiliations:** 1 Integrative Medicine Cluster, Advanced Medical and Dental Institute (AMDI), Universiti Sains Malaysia, 13200, Kepala Batas, Penang, Malaysia; 2 Environmental Research Group, Department of Chemistry, Faculty of Science, University of Malaya, 50603 Kuala Lumpur, Malaysia

**Keywords:** cloud point extraction, non-ionic silicone surfactant, atrazine, propazine, spectrophotometry, milk samples

## Abstract

A new cloud point methodology was successfully used for the extraction of carcinogenic pesticides in milk samples as a prior step to their determination by spectrophotometry. In this work, non-ionic silicone surfactant, also known as 3-(3-hydroxypropyl-heptatrimethylxyloxane), was chosen as a green extraction solvent because of its structure and properties. The effect of different parameters, such as the type of surfactant, concentration and volume of surfactant, pH, salt, temperature, incubation time and water content on the cloud point extraction of carcinogenic pesticides such as atrazine and propazine, was studied in detail and a set of optimum conditions was established. A good correlation coefficient (*R^2^*) in the range of 0.991–0.997 for all calibration curves was obtained. The limit of detection was 1.06 µg l^−1^ (atrazine) and 1.22 µg l^−1^ (propazine), and the limit of quantitation was 3.54 µg l^−1^ (atrazine) and 4.07 µg l^−1^ (propazine). Satisfactory recoveries in the range of 81–108% were determined in milk samples at 5 and 1000 µg l^−1^, respectively, with low relative standard deviation, *n* = 3 of 0.301–7.45% in milk matrices. The proposed method is very convenient, rapid, cost-effective and environmentally friendly for food analysis.

## 1. Introduction

Atrazine and propazine have been the most excessively applied herbicides over 40 years in preventing the existence of broadleaf weeds in desired crops. They are ubiquitous environmental pollutants in soil, water and food samples. Their use has caused great concern because of their mobility and solubility in water, they strongly sorb onto soil and exist in a small amount of milk. Moreover, the triazine family has been classified as a human carcinogen [1]. In the European Union (EU), content residues in milk and cream are not higher than 50 µg l^−1^ [2]. Because of these restrictions, analytical methods are required for monitoring the widespread distribution and it is highly desirable that these be environmentally friendly ‘green' analytical methods [3].

Generally, liquid–liquid extraction (LLE) and solid-phase extraction are widely used to extract triazine species from food analysis [4]. Although these methods offer high reproducibility and high sample capacity, they are time-consuming and labour-intensive. Furthermore, a large amount of hazardous organic solvents is used, which is hazardous to the operators and the environment [5]. Owing to these multiple disadvantages, solid phase micro extraction (SPME) was introduced. The main advantages of SPME extraction technique are user-friendliness and low requirement of organic hazardous solvents. However, the SPME can be expensive and/or time-consuming [6]. With the rapid development of sample preparation technologies, a few researchers have discovered the principles and advantages of cloud point extraction (CPE).

CPE follows the principles of ‘green chemistry' because it uses small amounts of non-toxic organic surfactants compared to toxic organic solvents [7]. CPE manipulates the temperature and concentration of surfactant to move the analyte into a micelle phase for separation. CPE is performed by adding a surfactant solution to the sample at levels exceeding the critical micelle concentration (CMC), allowing the formation of micelles. As the analytes dissolve and partition into the micelles, two immiscible isotropic phases form. The first one is the surfactant-rich phase, which contains the extracted analytes. The bulk of the aqueous phase is in equilibrium with the surfactant-rich phase. In CPE, temperature above the cloud point temperature (CPT) is used to induce the phase separation. The surfactant aggregate (a micelle) orients its hydrocarbon tails towards the centre to create a non-polar core. Isolated hydrophobic compounds (a large number of bioactive compounds) present in the aqueous solution are favourably partitioned in the hydrophobic core of micelles [8]. The use of non-ionic surfactant offers some advantages in the CPE for the extraction of the analyte compared to the toxic organic solvent. It has an ability to concentrate on the analyte with high recoveries. Besides, it is safe and cheaper; a very small amount of the relatively non-flammable and non-volatile surfactant is required.

Up to now, non-ionic surfactants (mainly polyoxyethylenenated alkyl phenols, from PONPE 7.5 and Triton series such as Triton X-100 and Triton X-114) are the most widely employed for organic compounds analysis with CPE. [9,10]. Triton X-114 is well known for micelle formation compared to other classes of non-ionic surfactant. In most cases, Triton X-114, as an extracting agent, was chosen as a surfactant owing to its high density of the surfactant-rich phase, relatively non-toxic reagent and low cloud point temperature, this facilitates phase separation by centrifugation. However, its aromatic chromophore has strong UV absorbance signals which become obstacles in the UV spectrophotometry detector. Therefore, a green non-ionic silicone surfactant, OFX 0309, is used to overcome this problem because it has more flexible polysiloxane chains without any aromatic structure. Furthermore, it can form more compact micelle structures which offer low water content in the surfactant-rich phase and are also low in density, thus, enhancing the extraction efficiency [11]. In addition, this surfactant plays an important role and is well known as a growing class of raw materials used in the cosmetic, food and pharmaceutical industries; its biocompatibility, safety to humans and environmentally friendly characteristics have been proved for a long time [12]. Moreover, the US FDA (Food and Drug Administration, USA) has permitted this OFX 0309 surfactant for internal consumption.

The aim of this study was to develop a simple and sensitive CPE method, coupled with spectrophotometry, for the determination of triazine species in milk samples using OFX 0309 as a green non-ionic silicone surfactant for the first time. The influences of main parameters, such as the types of surfactant, concentration and volume of surfactant, pH, salt, temperature, incubation time and water content on the extraction efficiency of triazine species (such as atrazine and propazine), were investigated and optimized in detail. Finally, figures of merit of the proposed method were compared with several reported methods in the literature.

## 2. Experimental procedures

### 2.1. Reagents and materials

Dow Corning OFX 0309 was purchased from Ingredients Plus, Malaysia. Atrazine (molecular weight: 215.68 g mol^−1^, *λ*_max_: 222 nm) and propazine (molecular weight: 229.71 g mol^−1^, *λ*_max_: 222 nm) were purchased from Dr Ehrenstorfer, Germany (99% purity). Standard stock solutions of atrazine (1000 mg l^−1^) and propazine (1000 mg l^−1^) were prepared in methanol. The working solutions were freshly prepared daily by an appropriate dilution of the stock solutions in deionized water. For all experiments, OFX 0309 surfactant and both triazine species were used without further purification. Hydrochloric acid (HCl) and sodium hydroxide (NaOH) were used for pH adjustment. Potassium carbonate (K_2_CO_3_), sodium carbonate (Na_2_CO_3_), potassium hydroxide (KOH), potassium chloride (KCl), sodium chloride (NaCl), sodium nitrate (Na_2_NO_3_) and sodium sulfate (Na_2_SO_4_) (purchased from QRec, Malaysia) were prepared by dissolving an appropriate amount in deionized water. Trichloroacetic acid (TCA), purchased from Fisher Chemical, USA, was used in the deproteination of milk samples.

### 2.2. Instrumentation

**Figure 1.**
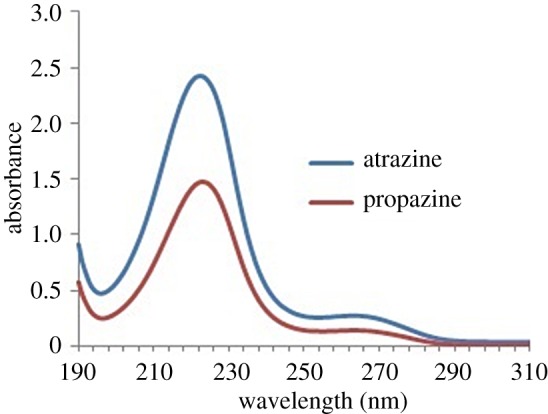
The absorption of UV–Vis spectra of triazine herbicides species.

A Perkin Elmer Precisely, Model Lambda 25 UV–Vis spectrometer (Massachusetts, USA) was used for the single measurement of the atrazine and propazine. A Memmert water bath, Schwabach, Germany was used and maintained at the desired temperature. The pH values of both triazine species solutions were determined by the pH meter (Hanna Instrument, USA). The absorption of UV–Vis spectra for atrazine and propazine is shown in figure 1.

### 2.3. Deproteination of milk

The bovine milk samples were randomly purchased from the local market in Bertam, Penang (Malaysia). The milk samples were freshly opened and stored at 4°C. The protein and fat of the milk samples, which might affect the analysis of the triazines according to the procedure adopted from [13], were removed. First, 0.4 ml of 15% of TCA was added to 1 ml of milk which was previously diluted with 5 ml of distilled water. The mixture was shaken for 30 s and centrifuged for 5 min at 4500 r.p.m. Then, the supernatant obtained was transferred and the spiked samples were analysed according to the method in §2.4. The spiked milk samples were obtained by adding certain amounts of triazines standard solution to the blank milk samples.

### 2.4. Cloud point extraction

**Figure 2.**
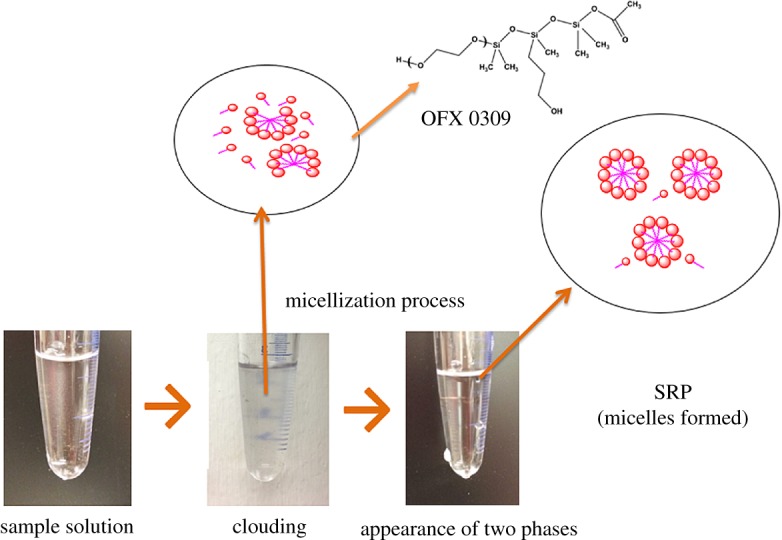
Schematic diagram of CPE. Conditions: 10 mg l^−1^ of triazine species, 0.4 v/v% OFX 0309 surfactant, 2.0 M of Na_2_SO_4_, pH 5, 50°C. SRP, surfactant-rich phase.

An aliquot of 1.0 ml of a sample or standard solution containing the appropriate amounts of analyte (10 mg l^−1^) and 1.0 ml of OFX 0309 (0.4 v/v%) were transferred into a centrifuge test tube. The pH of the sample solution was adjusted to 5 using 0.1 M of NaOH or HCl. To reach cloud point and formation of a cloudy solution, 0.5 ml of Na_2_SO_4_ (2.0 M) was added to the mixture. The solution was sonicated for 6 min and left to stand in a thermostatic bath for 15 min at 50°C. The appearances of two phases were obtained. The surfactant-rich phase at the top layer due to low density of surfactant was separated using a syringe to minimize the possibility of cross-contaminating the analyte with the corresponding aqueous phase. Subsequently, 2.0 ml of deionized water was added to the surfactant-rich phase to decrease its viscosity. It will also make the final volume feasible to be transferred into the optical cell for the measurement of each triazine species spectrophotometrically at the respective maximum absorption. Three replicated experimental data (*n* = 3) were collected in each optimization. The schematic diagram of the CPE method is shown in figure 2.

### 2.5. Water content

The water content in the surfactant-rich phase after the extraction was determined by drying the surfactant-rich phase at 353 K until no mass was observed in both CPE methods. The percentage of water content was obtained by calculating the weight difference of the surfactant-rich phase before and after drying. All the data given in this study were the average of triple measurement.

## 3. Results and discussion

The experiments were carried out to develop a simple and sensitive CPE method for a single spectrophotometric determination of the triazine species. The absorption spectra of each triazine species were recorded after showing a maximum absorption band at 222 nm. Therefore, all the measurements of the parameter studies were carried out at this wavelength. This was carried out in two separate analysis. The effects of various parameters on the performance of the method were investigated to achieve the highest sensitivity. The extraction efficiency is defined in equation (3.1):

3.1 $$\text{extraction efficiency}\;(\%)=\frac{C_{S}V_{S}}{C_{0}V_{0}}\times 100\text{\%},$$

where *C*_S_ represents the analyte concentration in the surfactant-rich phase volume *V*_S_*;* and *C*_0_ represents the analyte concentration in the initial sample–surfactant mixture of volume *V*_0_.

### 3.1. Optimization of non-ionic surfactant types

**Figure 3.**
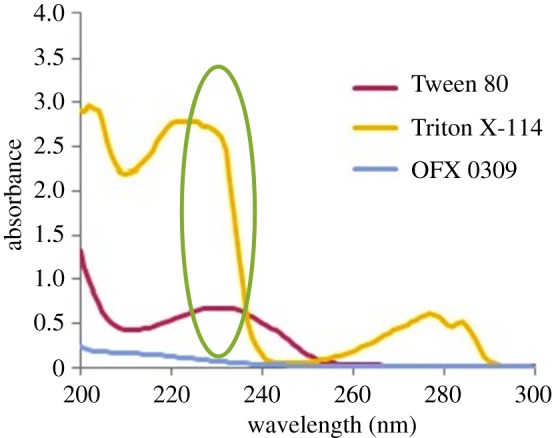
The screening absorption of UV–Vis spectra at 0.4 v/v% of non-ionic surfactants. Circled area shows the UV disturbance upon analysis of triazine species.

Generally, extraction is more efficient when more hydrophobic surfactants are used. Based on the literature review, three types of non-ionic surfactant have been selected for optimizing studies such as Triton X-114 [14], Tween 80 [15] and OFX 0309 as a new approach in the CPE. However, Triton X-114 was not further used in this study because of its aromatic chromophore structure with strong UV absorbance signals that become obstacles in UV detectors [16]. The absorbance signals are shown in figure 3. Meanwhile, Tween 80 was not preferable because of its viscosity. CPE will lose its sensitivity due to the high viscosity of Tween 80 surfactant [17]. Therefore, OFX 0309 was chosen to be studied in detail because it does not have any aromatic chromophore, which makes it more compatible to the UV–Vis spectrophotometry. In addition, the non-ionic surfactant of OFX 0309 has a low density and viscosity compared with Triton X-114 and Tween surfactants. Owing to its low density, the surfactant-rich phase was observed at the upper layer, which reduced the cross-contamination while extracting the surfactant-rich phase using a syringe for spectrophotometry analysis.

### 3.2. Effect of OFX 0309 surfactant concentration

**Figure 4.**
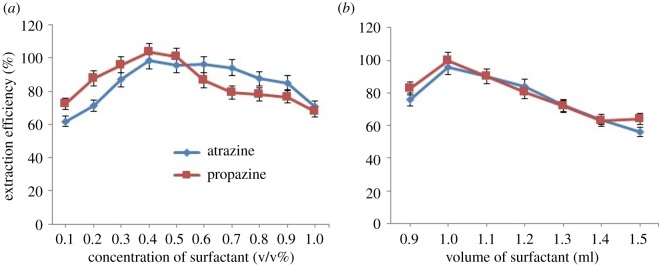
(*a*) Effect of concentration of OFX 0309 surfactant towards extraction efficiency of both triazine species. Conditions: 10 mg l^−1^ of triazine species, 2.0 M of Na_2_SO_4_, pH 5, 50°C. (*b*) Effect of volume of OFX 0309 surfactant towards extraction efficiency of both triazine species. Conditions: 10 mg l^−1^ of triazine species, 0.4 v/v% OFX 0309 surfactant, 2.0 M of Na_2_SO_4_, pH 5, 50°C.

The minimum concentration of surfactant used was desired in this work to obtain the maximum extraction of atrazine and propazine. However, the concentration of surfactant must be sufficient for the formation of micelle aggregates and quantitative extraction of the target analytes. There is a narrow range to achieve easy phase separation and maximum extraction efficiency. Beyond this range, the pre-concentration factor would decrease or the accuracy and reproducibility would most likely suffer [18]. The increase in surfactant concentration will increase the number of hydrophobic micelles and cause the increase in the extraction ability of surfactant, which also increases the extraction efficiency [19]. The surfactant-rich phase increased as the concentration of surfactant was increased to maintain both material balances. The effect of surfactant concentration on the extraction efficiency was evaluated in the range of 0.1–1.0 v/v% as shown in figure 4*a*. The extraction efficiency increased as the concentration was increased up to 0.4 v/v% and decreased as the concentration was increased up to 1.0 v/v%. This result might be related to the presence of high amount of OFX 0309 surfactant, resulting in an increase in the volume of the surfactant-rich phase. In addition, the viscosity of the surfactant-rich phase increased, leading to poor sensitivity determination of analysis. At lower OFX 0309 concentrations (less than 0.4 v/v%), the extraction efficiency of both triazine species was low, probably due to assemblies that were inadequate to quantitatively entrap the hydrophobic species [20]. Therefore, 0.4 v/v% was selected for further study.

### 3.3. Effect of volume of OFX 0309 surfactant

The volume of surfactant used in CPE is one of the parameters that affect the obtainment of high percentage of recoveries. Based on that, the volume of OFX 0309 surfactant was studied in the range of 0.1 to 1.5 ml. As shown in figure 4*b*, the percentage of recovery increased as the volume of surfactant was increased from 0.9 to 1.0 ml and decreased at a higher volume of OFX 0309 surfactant. This is because the analytical signal deteriorates due to an increase in the final volume and viscosity of the surfactant phase [21]. Below 0.9 ml of OFX 0309 surfactant, no separation of phase was observed. This might be because the surfactant molecules present were not enough to form micelles for entrapping the species [22]. Therefore, 1.0 ml of OFX 0309 surfactant was used as the optimum condition.

### 3.4. Effect of pH

**Figure 5.**
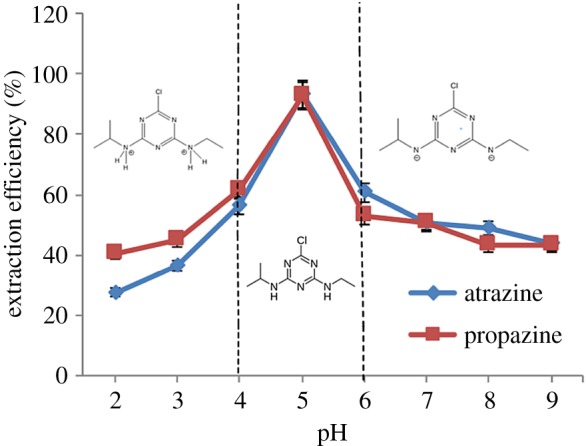
Effect of pH towards extraction efficiency of both triazine species. Conditions: 10 mg l^−1^ of triazine species, 0.4 v/v% OFX 0309 surfactant, 2.0 M of Na_2_SO_4_, 50°C.

The pH of the sample solution plays an important role in the extraction of analytes because the pH value can affect the existing form and partitioning of the analytes in the CPE [23]. In this work, the effect of pH 2 to pH 9 was studied. Figure 5 shows the relation between the pH and extraction efficiency for both triazine species (p*K*_a_ 1.7). The highest extraction recoveries were obtained at pH 5 for both triazine species, where the uncharged form of target analyte prevailed. Maximum extraction efficiency was obtained when the analytes could exist as neutral molecules [24]. The analyte, which is too acidic (pH < 5), will too easily degrade and get protonated [25]. In addition, the extraction efficiency increased because the triazine species would be distributed more into the surfactant-rich phase as their solubility in water became low [26]. However, the extraction efficiency for both species started to drop at pH more than 5. This is mainly because triazine species in the form of weak bases and strong acid or alkali environment were not beneficial to the formation of CPE. Therefore, pH 5 was selected as the optimum condition.

### 3.5. Effect of salt

Generally, phase separation in CPE can be carried out by heating the mixture containing the surfactant above the cloud point temperature. However, too high temperature might lead to analyte losses. Based on this, the salting-out effect was introduced as an alternative to induce the phase separation. Above CPT, an aqueous solution of the non-ionic surfactant micellar system has the ability to decrease its solubility and becomes turbid [27]. The presence of salt is known to decrease the CPT and increase the volume of the surfactant-rich phase. As the CPT is decreased, the extraction efficiency is increased [28]. Other than that, addition of salt is also known to decrease the analyte concentration in the aqueous phase [29].

**Figure 6.**
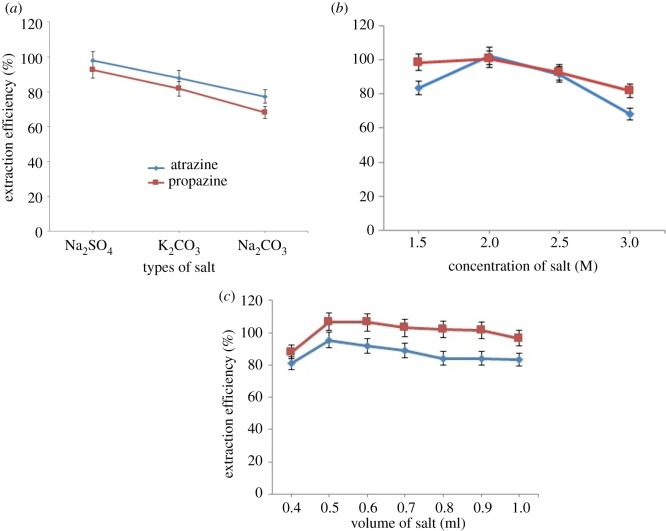
(*a*) Effect of types of salt towards extraction efficiency of both triazine species. Conditions: 10 mg l^−1^ of triazine species, 0.4 v/v% OFX 0309 surfactant, 2.0 M of salt, pH 5, 50°C. (*b*) Effect of salt concentration towards extraction efficiency of both triazine species. Conditions: 10 mg l^−1^ of triazine species, 0.4 v/v% surfactant, pH 5, 50°C. (*c*) Effect of salt volume towards extraction efficiency of both triazine species. Conditions: 10 mg l^−1^ of triazine species, 0.4 v/v% OFX 0309 surfactant, 2.0 M of Na_2_SO_4_, pH 5, 50°C.

In this work, the appropriate selections of salts to induce phase separation in CPE were investigated to improve the capability of phase separation in CPE. The salts studied were Na_2_SO_4_, K_2_CO_3_, Na_2_CO_3_, KOH, KCl, NaCl and Na_2_NO_3_. However, only three salts, Na_2_SO_4_, K_2_CO_3_ and Na_2_CO_3_, showed a two-phase separation of the solution. Some salts may act as a hydrogen bonding breaker and increase the cloud point (salting in), while some act as hydrogen bonding maker and decrease the cloud point (salting out) [30]. The good extraction efficiency was shown by Na_2_SO_4_. This is due to the kosmotropic ions $\left({\text{CO}}_{3}^{2-},{\text{ SO}}_{4}^{2-}\right)$ which have stronger interaction with water molecule than water itself. Thus, the ions are capable of breaking the water–water hydrogen bond and beneficial to the phase separation formation. ${\text{SO}}_{4}^{2-}$ ion is likely to cause the decrease in the self-association of water molecule. After comparing, Na_2_SO_4_ was chosen because the presence of Na^+^ cation may reduce the cloud point from the dehydration of polyethylene chain [15] and because ${\text{SO}}_{4}^{2-}$ is a polyvalent ion, it will cause faster dehydration from the polyethylene chain [31]. In addition, Na_2_SO_4_ salt increased the size of the micelles and aggregation number, thus enhancing the solubility of analytes in the surfactant-rich phase, so more water went to the aqueous phase [32]. As shown in figure 6*a*, the type of salt impacts the extraction efficiency of triazine species; the ability of the salt to enhance the recoveries of triazine species was in the order Na_2_SO_4_ > K_2_CO_3_ > Na_2_CO_3._

The presence of Na_2_SO_4_ shows a significant effect in lowering the CPT of surfactant and enhancing the extraction efficiency. The concentration of salt shows an effect in the CPE percentage of recovery because the addition of salt will enhance phase separation. As shown in figure 6*b*, different concentrations of salt were explored in this study ranging from 0.5 to 3.0 M. No significant enhancement in the phase separation was observed using 0.5 and 1.0 M of Na_2_SO_4_ as no phase separation was detected. The highest extraction efficiency was obtained by CPE with a salt concentration of 2.0 M, but above this concentration the extraction efficiency was decreased. This indicated that addition of a suitable salt reduced the solubility of the triazine species in the aqueous phase through a salting-out effect and decreased the ‘free water' concentration in the surfactant-rich phase. Consequently, the extraction efficiency improved as the salt concentration increased. However, if the Na_2_SO_4_ concentration is too high, the surfactant-rich phase becomes viscous, which makes it difficult to separate the surfactant-rich phase. Thus, 2.0 M concentration of Na_2_SO_4_ was chosen for further study.

The extraction efficiency and surfactant-rich phase volume were notably influenced by the volume of salt [33]. In this work, the volume of salt ranging from 0.1 to 1.0 ml was studied for both triazine species. However, below 0.4 ml no phase separation was observed. This phenomenon occurred because of the poor salting-out effect. Based on data shown in figure 6*c*, the extraction efficiency increased from 0.4 to 0.5 ml, while beyond 0.5 ml the efficiency started to decrease. The addition of salt above 0.5 ml compressed the volume of surfactant-rich phase because of the dehydration process and reduced the extraction efficiency. Therefore, 0.5 ml was selected as the optimum condition.

### 3.6. Temperature and incubation time

**Figure 7.**
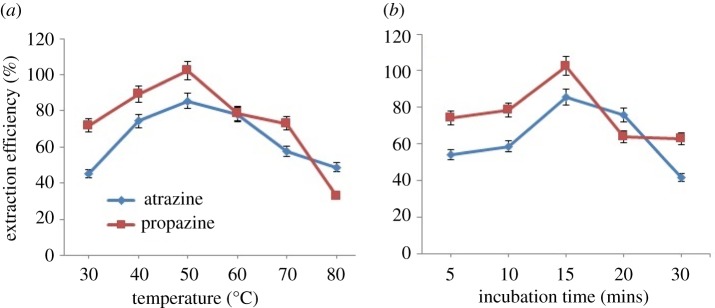
(*a*) Effect of temperature towards extraction efficiency of both triazine species. Conditions: 10 mg l^−1^ of triazine species, 0.4 v/v% OFX 0309 surfactant, 2.0 M of Na_2_SO_4_, pH 5. (*b*) Effect of incubation time towards extraction efficiency of both triazine species. Conditions: 10 mg l^−1^ of triazine species, 0.4 v/v% of OFX 0309 surfactant, pH 5, 2.0 M of Na_2_SO_4_, 50°C.

The effects of temperature on the extraction efficiency of triazine species are illustrated in figure 7*a* for both triazine species. The CMC of non-ionic surfactant decreased with temperature; while with the increase of temperature, the number of hydrophobic micelles in the surfactant-rich phase correspondingly became higher, causing an increase in the extraction ability of OFX 0309 surfactant towards triazine species due to dehydration in the external layer of micelles [34]. However, the excessively high temperature can also lead to the decomposition of analytes [35]. The increase of temperature will raise the viscosity of the surfactant-rich phase and result in a dilute problem with organic solvent [36]. Figure 7*a* shows evidence where the recovery percentages of both triazine species increased from 30 to 50°C, while beyond 50°C, the recovery percentages decreased due to the increases of viscosity.

CPE extraction is a type of equilibrium extraction. The optimal extraction efficiency was obtained once the equilibrium was established. The shortest incubation time was preferable in the CPE. Hence, the effect of incubation time on extraction efficiency was investigated in the range of 5 to 30 min. The experimental results shown in figure 7*b* indicate that the recovery of the triazine species decreased when the extraction time was longer than 15 min. The extraction equilibrium can be achieved within 15 min. This was probably because the contact surface area between the triazine species and extraction phase was very large due to the tiny drops of the upper extraction phase that formed evenly in the solution [37]. This may have happened due to the dehydration of salt molecules where the breaking of hydrogen bonding from water molecules increased the size of micelles and enhanced the solubility of the analytes [12]. The extraction of equilibrium can be achieved in a short time when the phase transfer of the target analyte species is fast [38]. Thus, the extraction time was set at 15 min.

### 3.7. Water content in surfactant-rich phase

**Figure 8.**
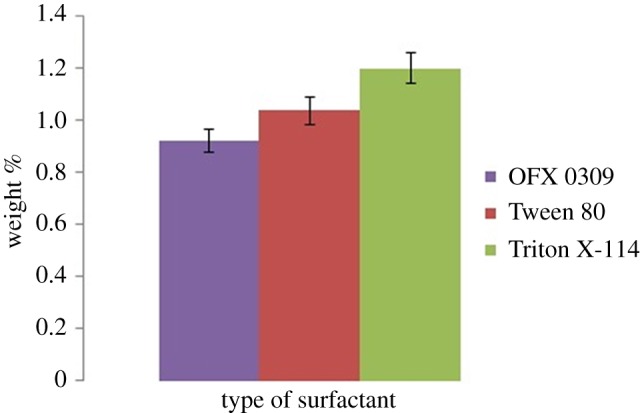
Water content in surfactant-rich phase obtained from CPE. Conditions: 10 mg l^−1^ of triazine species, 0.4 v/v% OFX 0309 surfactant, 2.0 M of Na_2_SO_4_, pH 5, 50°C.

The water content in surfactant-rich phase was studied to see which surfactant had the lowest water content. The performance of CPE was affected by the water content because in the surfactant-rich phase, it resulted in higher concentration of the analytes [39]. Data in figure 8 show that OFX 0309 has low water content in the surfactant-rich phase which is less than 1.0% compared to Triton X-114 and Tween surfactants. OFX 0309 molecules have the ability to make the arrangement of molecules more compact due to its flexible silicone chain structure [40]. The structure causes the micelles to be effectively compressed and with smaller spaces remaining with the water inside or among the micelles. The high flexibility polysiloxane chain with low cohesive energy of non-ionic surfactant offers more conformation which results in a compact micelle structure and low water content in the surfactant-rich phase [41].

### 3.8. Interference study

The selectivity of the method was investigated where 1 ml of the sample solution, containing 10 mg l^−1^ of atrazine species and 10 mg l^−1^ of Na^+^, K^+^, Cl^−^, ${\text{CO}}_{3}^{2-}$ and OH^−^, was extracted under the optimum experimental condition. The same procedure was carried out for propazine species. The results given in table 1 along with the recovery values reveal that there is no significant interference by the diverse ions present at moderate concentration. Ions were interfering when it caused variations greater than ±5%. The results confirm good selectivity of the proposed method and applicability of the method to the accurate determination of triazine species in milk sample.

**Table 1.** Interference study.

		extraction efficiency (%)	
ions	concentration (mg l^−1^)	atrazine	propazine
Na^+^	10	89	84
K^+^	10	89	81
Cl^−^	10	85	81
CO_3_^2−^	10	89	81
OH^−^	10	89	83

### 3.9. Evaluation of the method

#### 3.9.1. Analytical performance of the method

Under the optimized conditions, the calibration graph was obtained in the range of 5–2000 µg l^−1^ with correlation coefficient (*R^2^*) in the range of 0.991–0.997 for all the calibration curves. The calibration curve was prepared using the mean value of absorbance versus the triazine species concentration of three replicate experiments. The limit of detection (LOD) and limit of quantification (LOQ) were calculated using 3 *s*/*b* and 10 *s*/*b*, where *s* is the standard deviation of 10 replicate measurements of blank milk and *b* is the slope of calibration curve. The LODs calculated were 1.0 µg l^−1^ (atrazine) and 1.22 µg l^−1^ (propazine), and the LOQs were 3.54 µg l^−1^ (atrazine) and 4.07 µg l^−1^ (propazine).

#### 3.9.2. Application of proposed cloud point extraction method to milk samples

The proposed method of CPE has been applied in a recovery study with spiked samples at two different concentration levels to evaluate its accuracy. The recovery study was carried out in three replicates; (*n* = 3) tested at each concentration level. According to the results tabulated in table 2, the spiked concentration of triazine species can be quantitatively recovered from the milk samples by the proposed procedure. The recoveries for the addition of two different concentration levels, 5 µg l^−1^ and 1000 µg l^−1^ of triazine species in milk samples were in the range of 81–108% with relative standard deviation (RSD), (*n* = 3) of 0.301–7.45%. Based on the study, the matrix effect was not significant because only minimal components of milk were extracted in the surfactant-rich phase. Although atrazine and propazine were found in the blank sample, the concentrations detected were lower than the maximal residue limits of 900 µg l^−1^ established by the Food and Agriculture Organization of the United Nations and World Health Organization [42]. These results demonstrate the applicability of the proposed method for the measurement of triazine species in milk samples using spectrophotometry.

**Table 2.** Recovery of triazine species in spiked milk samples.

	atrazine			propazine		
samples	added (μg l^−1^)	^a^found (μg l^−1^ ± s.d.)	recovery (%)	added (μg l^−1^)	^a^found (μg l^−1^ ± s.d.)	recovery (%)
fresh milk	0	2.728	—	0	2.728	—
	5	3.81 ± 0.011^a^	102	5	3.57 ± 0.006	91
	1000	3.57 ± 0.024	81	1000	3.43 ± 0.106	89
low fat milk	0	2.728	—	0	2.728	—
	5	3.89 ± 0112	82	5	3.73 ± 0.278	94
	1000	3.82 ± 0.090	89	1000	3.69 ± 0.178	84
full cream milk	0	2.725	—	0	2.725	—
	5	3.66 ± 0.006	85	5	3.54 ± 0.004	86
	1000	3.58 ± 0.012	84	1000	3.66 ± 0.098	86
UHT milk	0	2.802	—	0	2.802	—
	5	3.85 ± 0.116	81	5	3.77 ± 0.115	108
	1000	3.65 ± 0.069	82	1000	3.89 ± 0.155	90
colostrum milk	0	2.930	—	0	2.930	—
	5	4.08 ± 0.066	81	5	3.86 ± 0.054	84
	1000	3.99 ± 0.267	83	1000	3.58 ± 0.026	87

^a^Mean ± s.d.

#### 3.9.3. Comparison with literature studies

The results of the proposed method and the reported methods were compared. Table 3 shows the comparison of performance between the proposed method with other reported CPE methods in the determination of triazine species for environmental and milk samples. There is only one reported research for the determination of triazine species in milk samples using the CPE method. The proposed method provides advantages such as lower LOD and LOQ compared with other reported CPE methods. Other than that, the proposed method gives satisfactory percentage of recoveries at a minimum concentration of surfactant by using this newly applied non-ionic silicone surfactant.

**Table 3.** Comparison of performance between the proposed method with other reported CPE methods for the determination of triazine species. n.a., not available.

samples	surfactant	determination	linearity (μg l^−1^)	LOD (μg l^−1^)	LOQ (μg l^−1^)	recovery (%)	ref.
water and soil	Triton X-114	HPLC-UV	16–10 000	water: 3.5 soil: 4.0	water: 11.0 soil: 13.0	water: 93–99 soil: 86–94	[43]
river water	Triton X-114	HPLC-DAD	1–50	0.1–0.3	0.4–0.9	86–132	[8]
milk	Triton X-114	HPLC-UV	50–2000	6.8–11.2	22.6–37.3	71–97	[1]
water, soil and vegetables	PEG-6000	HPLC-UV	1–10 000	water: 0.04–5.8 soil: 0.1–1.4 vegetables: 0.1–1.5	n.a.	water: 85–91 soil: 84–93 vegetables: 84–92	[44]
food	Triton X-100 and CTAB	UV–Vis	50–500	11.2–13.3	37.2–44.3	97–104	[45]
milk	OFX 0309	UV–Vis	5–2000	1.1–1.2	3.5–4.1	81–108	this method

## 4. Conclusion

In most cases, the high absorbance shown by many surfactants in the UV region prevents the use of the cloud point extraction coupled with the spectrophotometric method. In this paper, a new approach of non-ionic silicone surfactant, OFX 0309, was applied in CPE to eliminate the UV absorbance of surfactant in determining triazine species in milk samples. Furthermore, the concentration of surfactant used for this method was lower than that of other methods and has been proved capable of extracting lower concentration of triazine species in milk samples. In this way, this method has overcome the disadvantages of the CPE method coupled with spectrophotometry in food analysis. The developed method of CPE was applied for determining the triazine species in milk samples. The CPE procedure offers good extraction efficiency towards triazine species by using OFX 0309. The non-ionic silicone surfactant, OFX 0309, can be explored further for extracting other pollutants because it is less toxic and has low water content, which enhance the extraction efficiency. Therefore, the use of non-ionic silicone surfactant as a new approach to solvent extraction in CPE makes the extraction procedure greener and environmentally friendly for food analysis.

## Data accessibility

This article does not contain any additional data.
