# Correction to ‘Determination of carcinogenic herbicides in milk samples using green non-ionic silicone surfactant of cloud point extraction and spectrophotometry’

**DOI:** 10.1098/rsos.180777

**Published:** 2018-06-13

**Authors:** N. I. Mohd, M. Raoov, S. Mohamad, N. N. M. Zain

**Affiliations:** 1 Integrative Medicine Cluster, Advanced Medical and Dental Institute (AMDI), Universiti Sains Malaysia, 13200, Kepala Batas, Penang, Malaysia; 2 Environmental Research Group, Department of Chemistry, Faculty of Science, University of Malaya, 50603 Kuala Lumpur, Malaysia

*R. Soc. open sci.* **5**, 171500. (Published 11 April 2018). (doi:10.1098/rsos.171500)

The order of authors and the affiliation for M. Raoov are incorrect in the published paper. The corrected author list and affiliations are shown above.

